# Off-Set and Focus Effects on Grade 5 Titanium to 6061 Aluminum Alloy Fiber Laser Weld

**DOI:** 10.3390/ma11112337

**Published:** 2018-11-21

**Authors:** Giuseppe Casalino, Sonia D’Ostuni, Pasquale Guglielmi, Paola Leo, Gianfranco Palumbo, Antonio Piccininni

**Affiliations:** 1 Department of Mechanics Management and Mathematics (DMMM), Politecnico di Bari, Viale Japigia, 182, 70126 Bari, Italy; pasquale.guglielmi@poliba.it (P.G.); gianfranco.pAlumbo@poliba.it (G.P.); antonio.piccininni@poliba.it (A.P.); 2 Department of Engineering for Innovation, University of Lecce, Via per Monteroni, 73100 Lecce, Italy; sonia.dostuni@unisalento.it (S.D.); paola.leo@unisalento.it (P.L.)

**Keywords:** laser offset welding, titanium, Aluminum, fiber laser, intermetallic layer, optimization

## Abstract

Joining dissimilar metal alloys together has become a major issue in the welding industry since the rapid development of innovative and performing multi-materials products. In case, titanium and Aluminum alloys can be laser-welded using a placement of the laser beam aside the weld centerline, which is called off-set. The fused zone is deep and narrow and the reaction between titanium and Aluminum is limited to a thin interlayer, which improves mechanical properties. In this paper, the effect of focus and off-set distance of the laser beam on the weldability of grade 5 titanium to 6061 Aluminum alloy dissimilar butt weld are presented. The interlayer thickness was correlated to the process parameters and tensile behavior of the weld. The map of deformation showed different deformations of the two weld sides. The data coming from the metallurgical and mechanical characterization of the weld were analyzed to figure out the best off-set and focus combination in the range studied.

## 1. Introduction

Fusion welding dissimilar metals depends strongly on the metallurgical compatibility. In fact, several metallurgical and thermo-physical problems can arise due to structural phase transition during solidification and cooling of the welding cycle [1]. The formation of intermetallic compounds (IMC) layer affects the quality of the assembly. Poor chemical affinity and metallurgical compatibility can result in a non-homogeneous and brittle interface. This calls for the improvement of weldability of dissimilar metal welds through understanding and control of the IMC layer.

Some authors conducted chemical and mechanical analyses on several Al-Ti IMC to check their strength and ductility [2]. Others proposed diffusion welding of Ti and Al multi-laminated materials [3]. They showed that TiAl_3_ forms quite rapidly in the temperature range of 660–680 °C. In a study on friction stir welding (FSW) of Al-Ti, the tool pin was plunged in the Al-side. During the stirring action, the particles of Ti in the Al matrix elongated less than the surrounding Al, leading to the formation of cavities in the nugget zone [4]. Some difficulties were highlighted during FSW dissimilar butt welds because of their tendency to crack and to form grooves, especially due to the high tool rotation speed [5].

More researchers showed valuable results related to laser fusion brazing welding. With this method, the laser light irradiates the Aluminum that melts and wets the solid Ti surface. Limitation in the interfacial reaction, which produces the layer morphology and thickness, favors the initiation of cracks and reduces the mechanical properties of the joints [6]. Song et al. demonstrated the viability of the laser welding of grade 5 titanium to AA6061 by direct laser brazing without filler metal and groove. The right thickness of the interfacial layer can improve the mechanical properties of the weld but interfacial non-homogeneity, weld porosity and spatter defects reduce the joint quality [7]. The focalization of the laser beam on the Al side often resulted challenging and negatively affected the seam quality [8].

The fiber laser has demonstrated to be high performing at welding titanium alone [9]. High-speed full penetration fiber laser welding of Ti and Al lap joints was investigated [10]. Fiber laser can reduce the formation of thick IMC layer [11]. The fiber laser-cold metal transfer arc hybrid welding was used to join the Ti6Al4V Ti alloy and AA6061 Al alloy in butt configuration. Authors identified the setting of process parameters that maximized the mechanical properties of the welds [12].

As a variant to standard laser welding, the laser beam can focus onto the Ti side at a very short distance from the weld centerline without chamfering and filler material. It was called laser offset welding (LOW) [13]. The effects of the welding conditions on the IMC layer were studied by both optical and electron microscopes. The solidified interface formed from the Ti heat-affected zone and Al liquid or from Ti liquid and Al liquid interaction were studied as-welded and after post welding heat treatments (PWHT) performed at 350 °C and 450 °C for AA5754 and Ti6Al4V butt joints [14].

The same approach was used for magnesium and steel dissimilar welding [15]. LOW was compared with laser-arc hybrid welding with Aluminum and steel weld and it has proved to be viable [16]. Laser–arc hybrid welding of high-strength steel and Aluminum alloy joints with brass filler demonstrated that the braze welded joint fabricated without a Cu-Zn interlayer fractured at the Al-Fe IMC [17]. 

A preliminary investigation on grade 5 titanium alloy (Ti6Al4V) and 6061 Aluminum dissimilar welding using a disk laser studied the effects of laser power and welding speed and demonstrated the weldability of the two alloys by the LOW technique [18]. In this paper, the off-set position and focus height on the weldability of grade 5 titanium (Ti6Al4V) to 6061 Aluminum alloy were evaluated. The combination of off-set and focus can determine the heat transmission from the titanium to the Aluminum side and the modality of the interlayer formation. The data coming from the metallurgical and mechanical characterization of the weld were analyzed for optimization of the mechanical properties. The map of deformation displayed the different deformation behavior of the two sides of the weld. The interlayer thickness was correlated to process parameters and tensile properties. An optimum value for the interlayer thickness was found. 

## 2. Materials and Methods

### 2.1. Material Properties and Weld Configuration

The dissimilar assemblies between grade 5 titanium (Ti6Al4V) and 6061 annealed Aluminum alloy were realized in butt configuration. The size of the sheets was 100 mm length by 50 mm width by 2 mm thickness. Despite the significant difference in the tensile properties between the two materials, the weld was performed by adopting the same thickness for both plates. Such a choice was consistent with the main purpose of the investigation, which aimed to explore the effects of focus and off-set distance on the metallurgical and tensile properties of the weld. The chemical composition and physical properties of the base metals are listed in Table 1 and Table 2, respectively. 

Ti6Al4V alloy was supplied in mill-annealed conditions.

### 2.2. Set-Up of Welding System 

**Figure 1:**
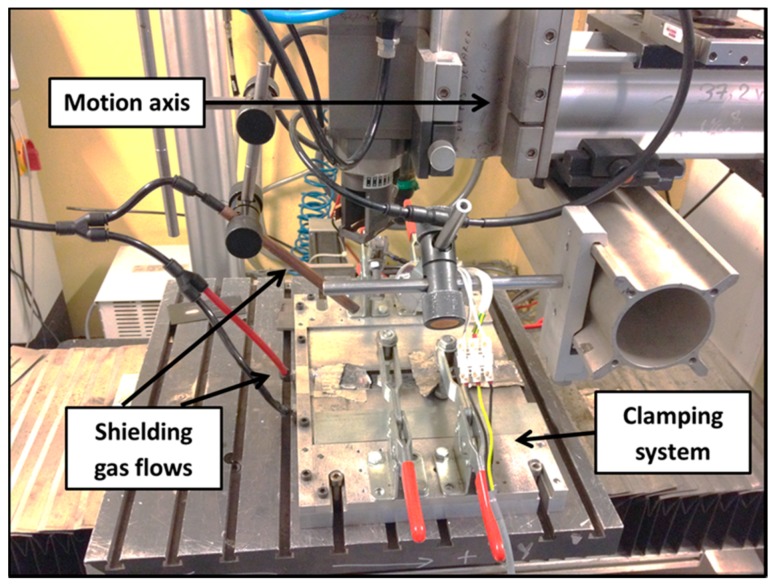
Experimental devices.

Integrated instruments were combined to accomplish the process. The whole apparatus set-up mainly comprises of a laser system with multi-axes machine, a shielding gas system and a workbench, equipped with clamps and supporting table (see Figure 1). An Yb:YAG disk laser operating at 1.03 µm, obtained with a 0.4 mm optical fiber and a maximum available power of 10 kW, was used in continuous wave regime. The beam parameter product (BPP) was 4 mm × mrad. Collimating lens and focusing lens with a focal length of 120 mm and 250 mm respectively were adopted for beam delivering. Preliminary tests were carried out to determine the focal plane and focal depth to ensure minimum spot diameter at high energy density. Then a beam profiler was adopted to detect the spatial intensity profile at the focal plane. A focus spot of about 400 μm diameter (1/e^2^ width) near-Gaussian distribution was positioned on the top surface of the titanium sheet. 

### 2.3. Plan of the Experiment

Preliminary bead-on-plate tests were conducted to find suitable values of process parameters and to identify key issues that could affect the repeatability of the process. The sheets were prepared by machining at low milling speed, grinding and cleaning with acetone to reduce the thermal contact resistance. The misalignment and gaps between the sheets were kept to a minimum. A camera inspected the gap between the sheets and it was found an average gap of about 35 µm, which provides for a good thermal coupling. An optimal clamping and an accurate sheet preparation could potentially minimize these concerns. High-precision cutting methods for interfaces and in situ monitoring of plates displacement could be adopted. However, in the industrial environment, the coupling between the sheets could be exasperated by other factors, including the length of the welds or the capability of the clamping system available. Furthermore, a greater accuracy in the coupling could increase the time and costs of the process. 

Therefore, the present study was conducted by adopting a common clamping system, and sheets were prepared according to standard mechanical procedures, in order to approximate the feasibility of the present technique for industrial purposes. 

**Figure 2:**
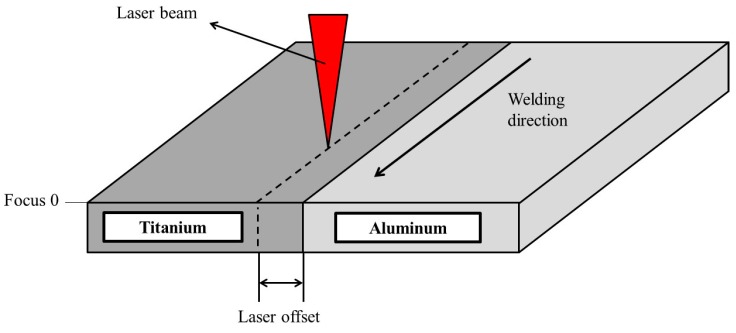
Offset and focus positions.

The welding speed was 2500 mm/min and the laser power 1500 W. Table 3 shows the 2-factors 3-levels experimental plan. The two variable factors were the off-set and the laser focus (see Figure 2).

Zero focus, i.e., minimum beam waist, was set at the plate surface. Negative values mean that the focus is below that surface.

## 3. Weld Metallurgy and Microhardness

Welds were cut perpendicular to the welding direction to characterize the transverse section. They were analysed by optical microscopy (OM; Nikon Epipkot 200, OM, Nikon, Tokyo, Japan)). The cross sections of the samples have been prepared using the standard metallographic grinding and polishing techniques and etched using Keller reagent (95 mL H_2_O, 2.5 mL HNO_3_, 1.5 mL HCl, HF 1 mL). 

The surfaces of the fusion zone (FZ), heat affected zone (HAZ), interlayer and grains size were evaluated using NIS-Element software for imaging analysis. NIS-Elements is a NIKON software (Nikon, Tokyo, Japan) supplied with Epiphot 200 OM. The software is tailored to facilitate image capture, object measurement and counting. 

The optical microscope analysis of the base material revealed the equiaxed recrystallized α grains and the β phase at the grain boundary [19]. The α phase is characterized by CBC (cubic body center) cell and it is stable from room temperature to 1066 °C (β transus temperature). At temperature higher then β transus the crystallographic structure changes in HCP (hexagonal close-packed) cell. The Vickers micro-hardness (HV_0.3/15_) of the alloy is equal to 290.3 ± 10.2.

AA6061 is a precipitation-hardenable Aluminum alloy, containing magnesium and silicon as its major alloying elements. In the Aluminium matrix, after chemical etching, Fe_3_SiAl_12_ grey outlined particles and Mg_2_Si dark particles [20] were observed. The Vickers micro-hardness (HV_0.3/15_) is 52.5 ± 1.15.

**Figure 3:**
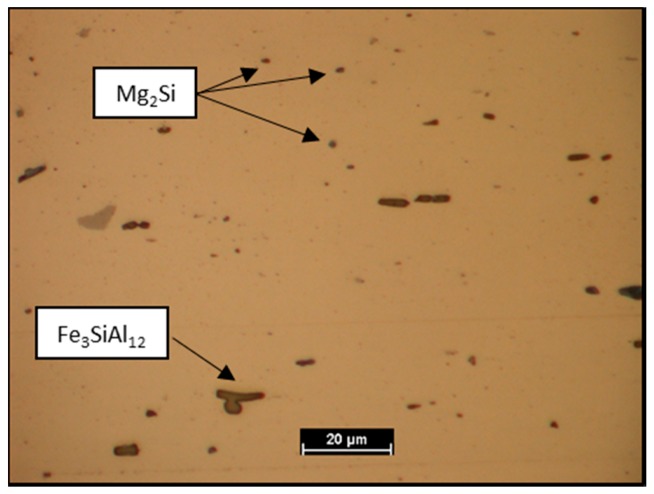
AA6061 as-received microstructure.

**Figure 4:**
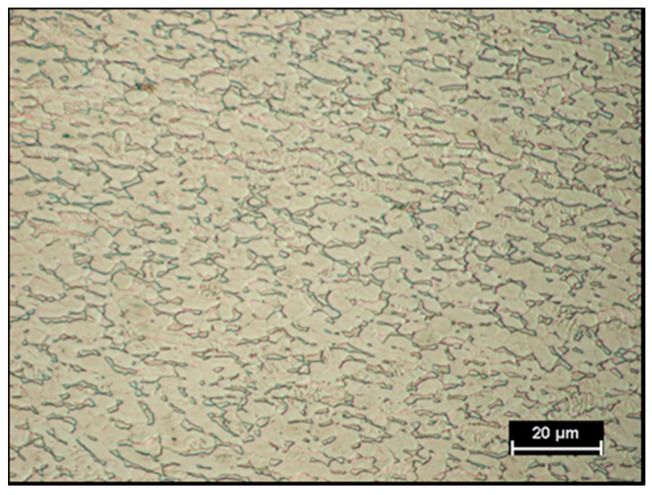
Grade 5 Titanium as-received microstructure.

In Figure 3 and Figure 4 the Aluminium and Titanium as-received microstructure are shown, respectively. 

**Figure 5:**
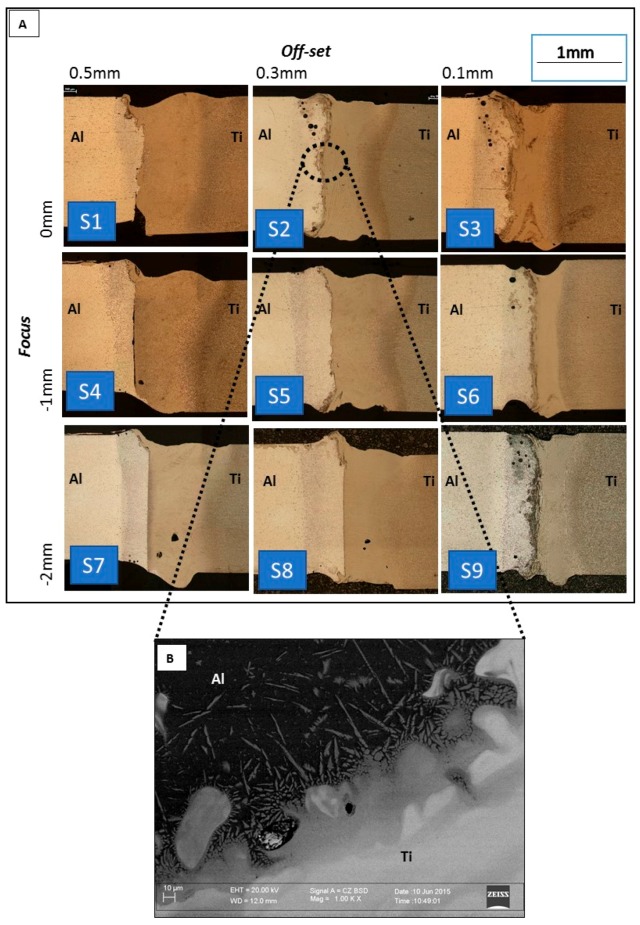
Cross sections (**A**) and SEM of IMC layer of S2 (**B**).

Figure 5A shows the cross section of the welds. For all the joints, the laser beam, which was focused on the Titanium side, produced the keyhole. The heat was conducted through the Ti-Al interface and generated the fusion of the Aluminum alloy.

Therefore, all the joints exhibited one fused zone for the al side and another for the Ti side, which were separated by the IMC layer. Particularly, in Figure 5B is shown a Scanning Electron Microscope (SEM) micrograph of the IMC layer in the highlighted circle of S2 sample (Figure 5A). As shown by P. Leo et al. [14], for dissimilar Aluminium Titanium joints, the composition of IMC layer is not uniform, varying from Al_3_Ti to Ti_3_Al as the distance from Aluminium fusion zone increases. The mechanical properties are not uniform along the thickness of the layer but the hardness increases moving from Al_3_Ti to Ti_3_Al. From the literature, it is known that the capillary cavity diameter, during the laser welding in the keyhole mode, is close to that of the laser spot [21]. In S3, S6 and S9 joints, processed with an offset of 0.1 mm, the keyhole in the Titanium extended to the Aluminum side and the fusion of Aluminum was generated also by direct effect of laser beam. In those joints a larger presence of defects can be observed. 

The Aluminum fusion zone exhibited a fine dendritic microstructure, characterized by inter-dendritic segregation of low-melting compounds. Particularly, columnar grains grew from the HAZ/ZF interface towards the Ti side. The growth of columnar grains, promoted by steep heat gradient, occurred in the opposite direction of the heat flow (perpendicular to the interface) [22,23,24]. 

**Figure 6:**
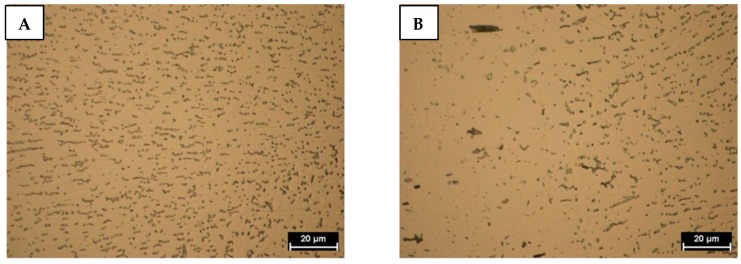
Aluminium microstructure in the fused zone (**A**) and fused/heat affected transition zone (**B**).

Under optical microscope light field observation, the heat affected zone did not exhibit evident microstructural changes (Figure 6). In fact, in the HAZ of the Aluminum alloy, coarsening or reversion of soluble second phases particle can happen. The last microstructural evolution (reversion) being more significant as the distance from fused zone is reduced where the thermal cycle is characterized by higher peak temperature. Coarsening and/or dissolution cannot be highlighted from optical analysis of the microstructure in Figure 6.

**Figure 7:**
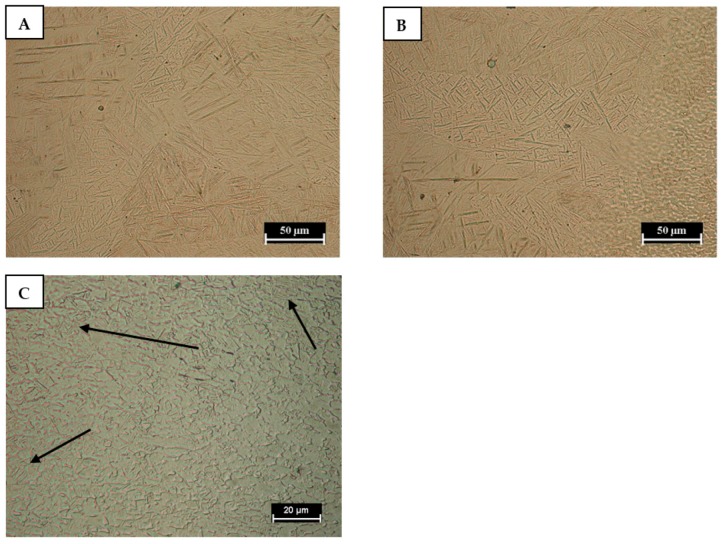
Titanium microstructure in the fused zone (**A**) fused/heat affected transition (**B**). Higher magnification of HAZ showing microstructural evolution respect to BM (**C**).

Titanium fuse zone microstructure (Figure 7A) was formed predominantly by acicular α′ martensite [25] and it was characterized by columnar grains. According to the literature [26], the microstructure of the laser-welded Ti6Al4V joints in keyhole mode tended to be completely martensitic due to the high cooling rates from the β field. The FZ/HAZ interface is shown in Figure 7B, while Figure 7C is a higher magnification micrograph of HAZ in order to highlight the microstructural features. In the HAZ, the α/β transformation on heating thermal cycle is only partial leading a lower amount of martensitic microstructure respect to the FZ, as shown by the arrows in Figure 7C, which refer to some martensitic areas in HAZ. In Figure 7C many untransformed grains can be observed. Especially, in the HAZ, the amount of β phase increases with temperature. Hence, the volume fraction of β phase is highest close to the fused zone. Upon cooling that β phase transforms mainly to martensite because of the high cooling rate induced by laser beam welding. So, being the volume fraction of β phase lower as distance from FZ increases, therefore, also the volume fraction of hardening phase due to β phase evolution on cooling decreases with the same trend.

Vickers microhardness profiles (0.3/15 s) were collected using a Vickers Affri Wiky 200JS2 microhardness tester (OM, Nikon, Tokyo, Japan) at half of weld cross section thickness. Distance between indentations was equal to 300 μm.

**Figure 8:**
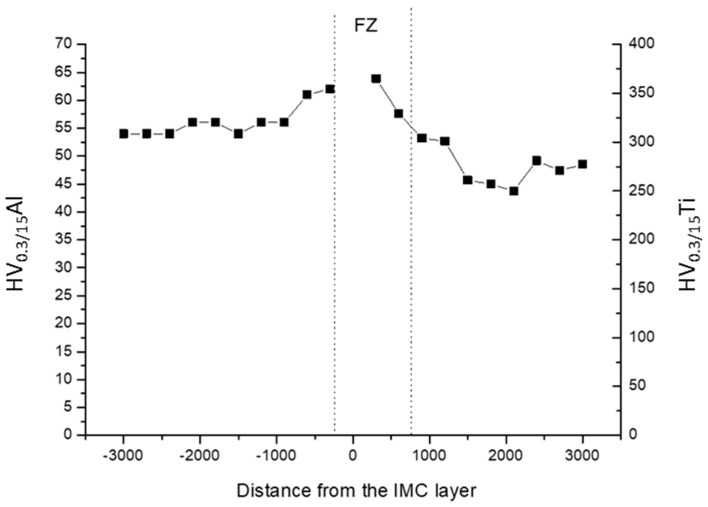
Microhardness profile and table for sample S8.

All the joints exhibit the same trends of hardness both in the fusion zone and the heat affected one. Figure 8 shows the microhardness profile in the transverse section of S8 weld at half the thickness. The microhardness was very high in the titanium FZ where the microstructure was martensitic. In the HAZ, the value diminished because the lower amount of the martensitic microstructure.

If compared with the hardness of Al base material, an increasing of hardness was observed both in the Al FZ and HAZ. In the FZ of Aluminum the hardening was due to the rapid cooling that produced a very fine solidification structure and solid solution strengthening. The increment of hardness in the HAZ of Aluminum could be due to the dissolution of coarse and soft magnesium compounds during the welding thermal cycle, being the alloy supplied in annealed condition [21].

Table 4 contains measured microhardness and relative means, which were evaluated along the joints thickness at 300 µm from the interface for both Al and Ti side. Those values come from three measurement in each side of the joint, respectively at 500 µm from the top and the bottom surfaces and at half thick of the joints (center point). 

It was found that average microhardness increased in the Ti side as the laser offset reduced at fixed focus height, see for instance the hardness values at the extreme value of laser offset when focus beam is at the top surface (S1 vs. S3) and at the bottom surface (S7 vs. S9). In fact, as laser offset became shorter, a smaller grain size formed in Ti fusion zone due to its small size (Table 5) and higher cooling rate. At fixed offset, also the average hardness along the thickness exhibited sensitiveness to defocusing. Defocusing of laser beam impose a variation of power density [27]. A microstructural evolution related to different welding thermal cycles can be induced in the joint. In the Ti side at laser offset equal to 0.5 mm, the hardness values along the thickness at the top, center and bottom points, were quite uniform at the extreme values of defocusing (S1 and S7 joints). On the contrary, S2 and S8 joints, as well as S3 and S9 ones, exhibited a strong variation in hardness values along the thickness (as confirmed by the values of standard deviation). Further works may assess the role of defocusing on microstructure evolution and hardness of the Ti side. The effect of focus height is more evident in the softer Aluminum side. In fact, the local values of hardness were higher for the points close to the focus position (top values in Table 4 for S1–S3 and bottom values for S7–S9). On the contrary, the joints S2, S5 and S8, in which the focus point was at a half of thickness, did not exhibit any trend in the local hardness values. The average hardness did not change significantly in the Al side even if the hardness was slightly higher when the laser beam focus was at the top surface (see S1–S3 weld). 

The average thickness of the interlayers was calculated as the ratio between the IMC area and the length of the layer. The IMC thickness was influenced by the laser offset value and varied between 4.44 and 89.38 µm (Table 5). 

IMC thickness increased with decreasing value of laser offset. On the other hand, lowering the focus Z at fixed offset caused the reduction of the IMC thickness [28]. 

## 4. Tensile Test

Tensile tests were carried out at room temperature using a 200-kN electromechanical tensile test machine (INSTRON, Norwood, Massachusetts USA), setting the crosshead speed to 1 mm/min. 

**Figure 9:**
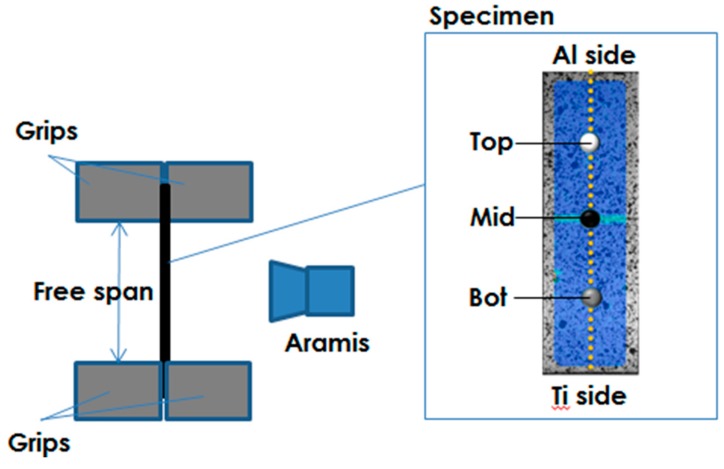
Scheme of the set-up adopted for the tensile test.

Strips with parallel sides were used as specimens and a constant free span length of 50 mm was adopted for all tests. The strain distribution over the specimen was acquired during the test by a non-contact Digital Image Correlation system (ARAMIS by GOM). The specimen was painted to create a stochastic pattern and two cameras allowed to acquire images with a 5 Hz frequency. In Figure 9, the set-up adopted in the experimental trials is presented. 

Material data concerning both the whole specimen and local areas could be obtained. Elongation at break (El%) and Ultimate Tensile Strength (UTS) were measured as the ratio between the maximum stroke and the free span and the ratio between the maximum load and the undeformed section of the specimen (S_0_), respectively. 

Local strain data from the Digital Image Correlation (DIC) system (i.e., data concerning the sample in the unaffected zone, in the HAZ and in the FZ) were also obtained from each tests; in addition, combining load data and local strain data from the DIC system, for the specimens which exhibited high enough strains, it was possible to obtain the flow stress curves in different locations: Top (in the region of Al), Bot (in the region of Ti) and Mid (in the region of Al altered by the presence of IMC). 

Results from tensile tests are showed in Table 6 in terms of data concerning the entire specimens (thus considered as composed by a unique material). It is worth mentioning the strict relationship between the elongation at fracture and the UTS. 

**Figure 10:**
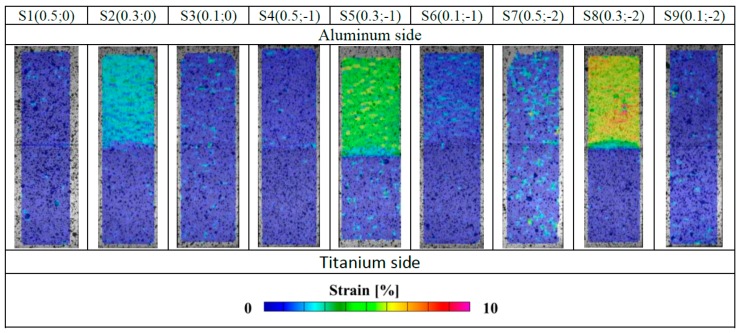
Deformation maps obtained by means of the DIC system (off-set; focus).

Because of the presence of dissimilar materials composing the joint, local analyses have been also performed. In Figure 10, deformation maps obtained by means of the DIC system have been presented: it is worthy of notice that quite a larger (and homogenous) strain level was reached in the Al side of joints obtained setting an off-set value equal to 0.3 (S2, S5 and S8).

**Figure 11:**
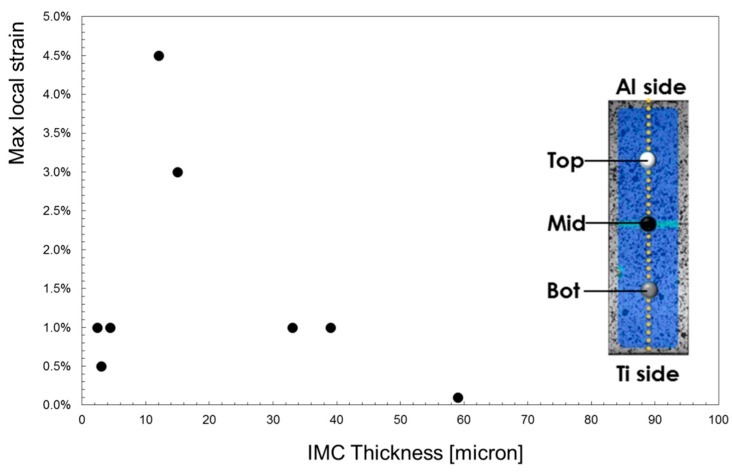
Strain levels reached in the region of the IMC layer.

It is noteworthy that a larger strain level was reached at midpoint (i.e., the region where the connection between the two materials is located): in such a region the value of the strain was lower than 1% in all specimens but not in the samples S5 and S8. To highlight the different local material behavior, in Figure 11, the maximum local strain in the mid-point has been correlated with the IMC thickness. It is noteworthy that for some value of the IMC layer, the level of the strain the material in the mid region can undergo reaches the highest values.

Focusing attention on the local flow stress curves, it may be noted in Figure 11 whose data concerning the joint obtained setting the offset to the intermediate value of 0.3 mm have been plotted, that the Mid-point region (where the IMC layer is located) has a strength higher than the Al one. Such a result agrees with microhardness measurements in Figure 8.

**Figure 12:**
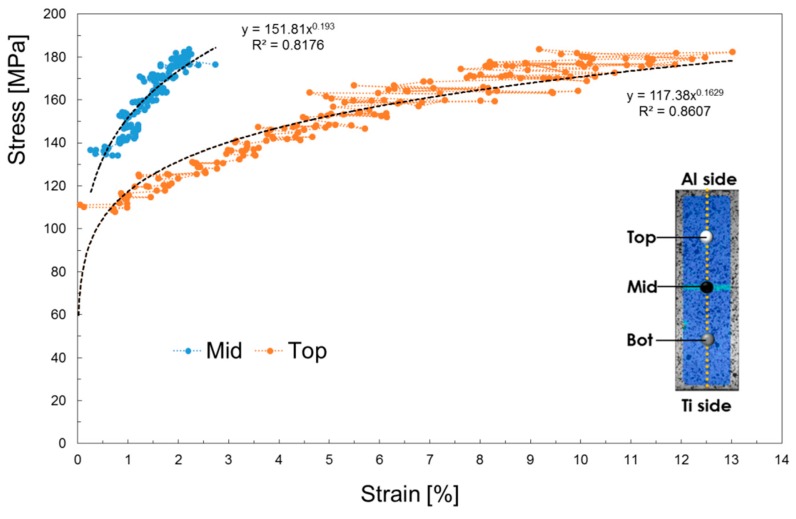
Local flow stress obtained from the test conducted on the sample welded setting the Off-set to 0.3 mm and the Focus to −2 mm.

Finally, Figure 12 shows the local flow stress obtained from the test conducted on the sample welded setting the Off-set to 0.3 mm and the Focus to −2 mm.

## 5. Analysis and Optimization of Tensile Properties

**Figure 13:**
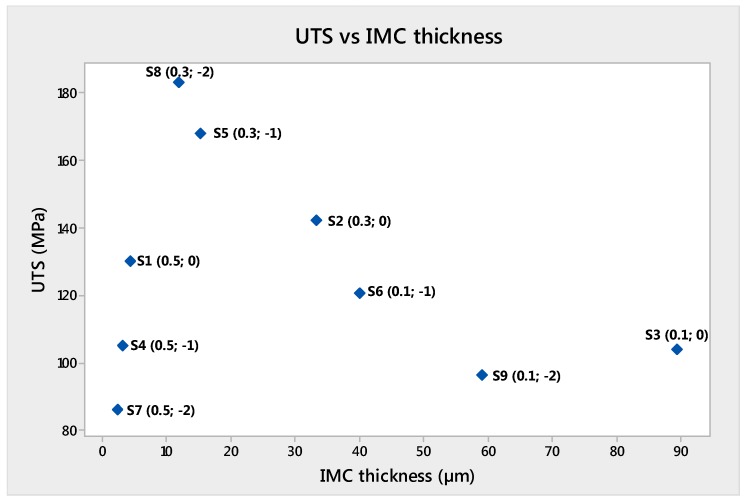
UTS versus IMC thickness.

**Figure 14:**
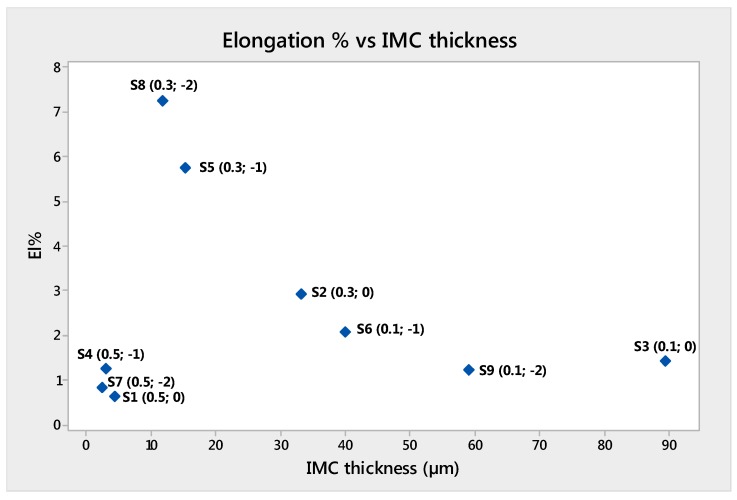
Elongation (%) versus IMC thickness.

Table 7 shows an at-a-glance the off-set level and the corresponding output measurements. UTS and E were plotted versus IMC thickness in Figure 13 and Figure 14. The analysis of these plots demonstrated that the best UTS and E were obtained with sample S8. S8 sample had 11.82 µm IMC thickness. Larger and lower values for the IMC thickness produced lower tensile properties. 

Sample S8 was processed using a 0.3 mm off-set and −2 mm focus. Larger and smaller off-set produced lower tensile properties. 

**Figure 15:**
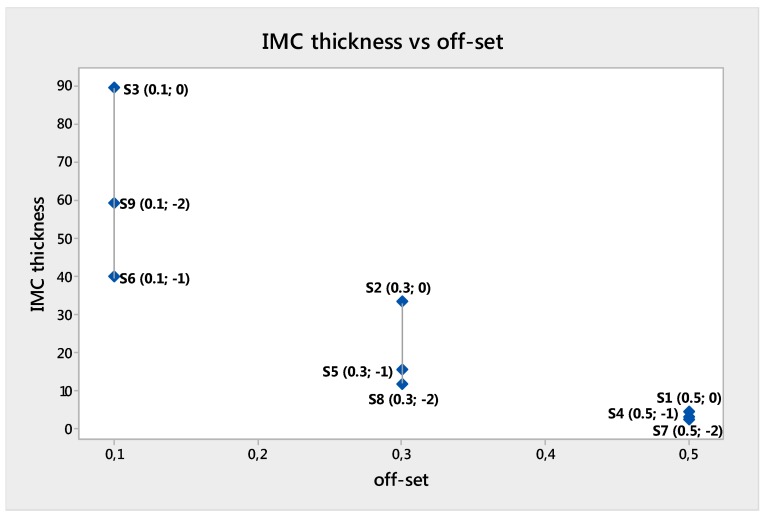
IMC versus off-set.

Samples S5 and S8 had 11.82 and 15.33 µm IMC thickness, which seem to be an optimal range for the IMC layer thickness for weld mechanical properties (Figure 15).

## 6. Conclusions

In this paper, the off-set position and focus height on the weldability of grade 5 titanium (Ti6Al4V) to 6061 Aluminum alloys were evaluated by experimental analysis. The collected experiments were evaluated in terms of metallurgical, mechanical, and optimization of mechanical properties.

The metallurgical characterization showed the formation of the classic IMC between Aluminum and titanium. However, the sensitiveness of the interlayer thickness on the process parameters was demonstrated. The laser off-set distance had influence on the thickness of the interlayer compounds.

The average hardness evaluated along the thickness usually increases in the Ti side at fixed focus length as the laser offset is reduced and, moreover, at fixed laser offset, exhibit some variations with defocusing. The process parameters influence slightly the average hardness in the Al-side, which is higher close to the focus position.

The UTS of welds varied from 86 to 183 MPa, and the elongation from 1 to 7%. The deformation was mainly located on the Aluminum side of the weld. The best UTS and elongation were obtained from the sample S8, whose off-set was 0.3 mm. The second best UTS and elongation were those of sample S5. That outcome confirmed the importance of the laser off-set and the interaction with the laser focus. Eventually, the optimal value for laser off-set was 0.3 mm.

For these reasons, it can be concluded that the formation of brittle compounds is unavoidable, the IMC thickness plays a key role in the weldability of grade 5 titanium (Ti6Al4V) to 6061 Aluminum alloys. IMC as thick as more than 15 µm enhanced the weld brittleness and lowered the mechanical strength. 

## Figures and Tables

**Table 1:** Chemical composition of Titanium grade 5 (weight %).

Ti	C	Fe	N	Al	O	V	H	Y	Other
**balance**	0.08 max	0.03 max	0.05 max	5.5–6.75	0.20 max	3.5–4.5	0.015 max	0.005 max	0.40

**Table 2:** Chemical composition of AA6061 (weight %).

Al	Si	Fe	Cu	Mn	Mg	Cr	Zn	Other	Other
**balance**	0.40–0.80	0.70 max	0.15–0.40	0.15	0.8–1.2	0.04–0.35	0.25 max	0.05 max	0.15 max

**Table 3:** Off-set and laser focus are in mm.

	S1	S2	S3	S4	S5	S6	S7	S8	S9
**Off-set**	0.5	0.3	0.1	0.5	0.3	0.1	0.5	0.3	0.1
**Focus**	0	0	0	−1	−1	−1	−2	−2	−2

**Table 4:** Micro hardness values along the thickness (see Table 3 for process parameters).

Sample	HV_0.3/15_ Top Al Side	HV_0.3/15_ Center Al Side	HV_0.3/15_ Bottom Al Side	HV_average_ Al Side	HV_0.3/15_ Top Ti Side	HV_0.3/15_ Center Ti Side	HV_0.3/15_ Bottom Ti Side	HV_average_ Ti Side
S1	71	65	68	68 ± 3	360	350	345	352 ± 8
S2	73	63	63	66 ± 6	407	354	381	381 ± 26
S3	78	64	70	71 ± 7	442	412	407	420 ± 19
S4	68	62	62	64 ± 3	357	340	352	350 ± 9
S5	66	64	70	67 ± 3	365	354	350	356 ± 8
S6	54	67	68	63 ± 8	359	410	358	376 ± 30
S7	60	65	68	64 ± 4	365	344	358	356 ± 10
S8	58	62	69	63 ± 6	400	365	377	381 ± 18
S9	59	59	64	61 ± 3	390	431	380	400 ± 27

**Table 5:** IMC layer thickness and fused zone dimensions (see Table 3 for process parameters).

Sample	IMC Thickness [µm]	FZ Area [mm^2^]	FZ Al Area [mm^2^]	FZ Ti Area [mm^2^]
S1	4.44	1.82	0.38	1.44
S2	33.24	2.17	1.00	1.17
S3	89.38	2.01	0.98	1.03
S4	3.12	2.47	0.79	1.68
S5	15.33	2.60	1.19	1.41
S6	59.10	1.86	0.95	0.91
S7	2.43	2.48	0.87	1.61
S8	11.82	2.71	1.31	1.40
S9	39.98	1.53	0.87	0.66

**Table 6:** Results from the tensile test (see Table 3 for process parameters).

Sample	Max Load [N]	UTS [MPa]	E_max_ [%]
S1	2.416	130	0.64
S2	4.311	142	2.92
S3	3.388	104	1.42
S4	3.178	105	1.26
S5	6.072	168	5.76
S6	4.215	120.7	2.08
S7	2.851	86	0.84
S8	6.201	183	7.26
S9	3.259	96.4	1.24

**Table 7:** Off-set levels and the corresponding output measurements (see Table 3 for focus levels).

Sample	Off-Set [mm]	IMC Thickness [µm]	UTS [MPa]	FZ Ti Area [mm^2^]
S1	0.5	4.44	130	0.64
S4	0.5	3.12	105	1.26
S7	0.5	2.43	86	0.84
S2	0.3	33.24	142	2.92
S8	0.3	11.82	183	7.26
S5	0.3	15.33	168	5.76
S3	0.1	89.38	104	1.42
S9	0.1	59.10	96.4	1.24
S6	0.1	39.98	120.7	2.08
